# Supplementary material for: Atrial Fibrillation and the Risk of Ventricular Arrhythmias and Cardiac Arrest: A Nationwide Population-Based Study
Source: J Clin Med. 2023 Jan 30;12(3):1075. doi: 10.3390/jcm12031075 (PMC9917986; doi:10.3390/jcm12031075)
Supplement: Supplementary file 1 [file jcm-12-01075-s001.zip › jcm-2140004-supplementary.pdf]

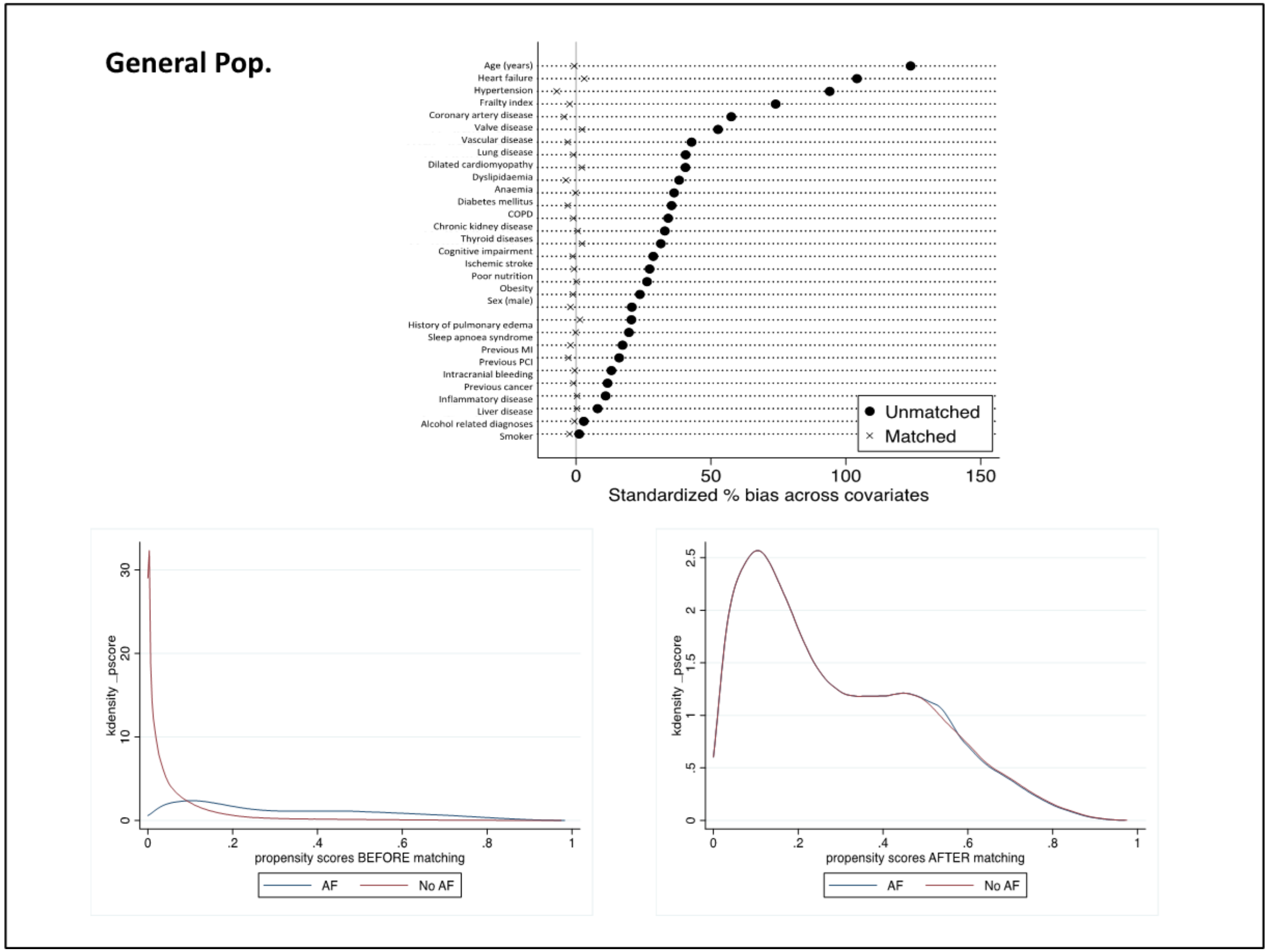

Supplemental Figure S1. Patients seen in French hospitals in 2013: Standardised percentages of bias across main baseline characteristics in unmatched and matched patients with AF and no AF (top panel) and propensity score distribution for unmatched (left lower panel) and matched (right lower panel) populations of patients with AF and no AF. Abbreviations: AF – atrial fibrillation
